# Observations on Fractures; and the Good Effects of Vesicatories in Promoting Union in Cases of Fracture Which May Not Have United Naturally. Likewise, an Improved Method of Removing Enlarged Tonsils, and Also of Cupping

**Published:** 1814-12

**Authors:** Richard Walker

**Affiliations:** Oxford


*For the Medical and Physical Journal.*

### *Observations on Fractures; and the good Effects of Vesicatories in promoting Union in Cases of Fracture which may not have united naturally. Likewise, an improved Method of removing Enlarged Tonsils, and also of Cupping.* By Mr. Richard Walker, of Oxford.

THE principal aim in the management of fractures should be, of course, to leave the limb, after the cure, or union of the bone, as nearly as possible in its natural state, respecting form, &c. this being essential to a due strength in the limb afterwards, as well as for appearance.

Perfection in this point, I well know, is not to be expected in a young surgeon commencing practice, whatever may have been his previous instructions, and observations upon others; and the object of the following observations will be to point out briefly the method of effecting a better cure than I have observed in the management of others.

It may be proper to observe, however, that in certain fractures, viz. such as are called *compound,* or *complicated,* although much may be effected by superior skill in the management of them, yet some degree of deformity, or irregularity, in the limb, it may be impossible to avert. In simple fractures, likewise, if the bone be broken *very obliquely,* some small vestige of the injury may be expected; but in a *transverse fracture,* especially in the leg, not connected with the joint, any vestige whatever of the injury, after the union of the bone, except an incidental thickening at the part, as sometimes happens at first, from a *redundancy of callus,* but which is in time absorbed, may be considered as reflecting disgrace on the surgeon.

I have attended to the reduction and after-treatment of not less than *a thousand fractured limbs,* simple and compound, assisting at most of them; and had the entire care, from beginning ginning to end, of nearly one-fourth of that number. The ordinary method was, to place them *on the side*, the joint being in a relaxed or bent position throughout the cure. Having observed, under the management of others, as well as my own, that, notwithstanding every possible attention to fractured legs, by this method, it not unfrequently happened, that, in the end, the tibia bowed more or less forward at the part broken, in all future cases, of which I had the entire care, which were many, I uniformly adopted the following method. I placed them at first on the side, the knee-joint being somewhat bent, and when the tension had completely subsided, by the usual discutient means, which, in simple fractures, was in about ten days, I turned them carefully round *on the heel*, the patient, of course, being at the same time turned round carefully, so as to lie directly on the back. The limb was now placed in a fracture-box, the knee being kept in a flexed position, by the adjustment of pillows, &c. with the usual appendages of splints, &c. By this method, I mended the broken legs so well, that I could defy an observer to point out which leg had been broken.

It might appear to be improper to turn the leg after it has been *set* as it is called, but an adept in these matters well knows, that, from the restlessness of the patient, and other causes, the limb requires occasional adjustment for a considerable time after its being first properly reduced, especially if laid on the side. My reason for placing the leg at first on the side, was, because the tension appeared to be less, and sooner subsided, in that position, and no mischief produced by afterwards moving it.* In compound fractures of the leg, I commonly placed them in the fracture-box from the beginning, having constantly found this method of

---

* Much attention and care is required, of course, in thus altering the position of the limb and body, without the fractured part being in the least disturbed; but, that it may be done with perfect safety at the time, and with eminent future advantage to the patient, I have had the experience of some scores of cases to confirm the truth of. I took the care of the fractured part myself, and by sufficient assistance contrived that the limb and body should be moved together in such perfect unison, that the fractured part remained as unmolested as if it had not been moved.

The coalescence of the ends of a broken bone is progressive, and, whilst the coalescence is going on, *yielding*, especially in the early part of it; hence admitting of adjusting, if required, during the early part of that process of nature.

In very old persons, it may be adviseable *not* to adopt this mode of practice; but to place the limb in the fracture-box at first.

placing the leg essential to the comfort of the patient and welfare of the limb.

It sometimes happens, notwithstanding the utmost attention and skill of the surgeon, that simple fractures do not unite, or coalesce, but remain loose as at first. This circumstance, I think, happens in legs chiefly, and in elderly persons. This is so rare an occurrence, that I do not recollect above six or eight instances of it. After various means tried in vain to effect a union, none succeeded but the dreadful resource of having the bone denuded, and the two ends sawed off. One patient, an elderly man, under this dilemma, was doomed to amputation: I proposed to the surgeon, (after every thing that had been suggested was tried in vain, and amputation had been proposed to the patient,) with a view to excite adhesive inflammation, to try *vesicatories;* this was complied with, and, by a repetition of blisters, applied round the limb, union of the bones was effected, and the man at this time (the circumstance having happened seventeen or eighteen years ago) has two good legs to walk on. There are some instances, as I have witnessed, of the union of the bones being prevented, by the intervention or formation of a membranous substance between the bones; in this case, of course, the operation before-mentioned must be resorted to. The latter cause may be suspected to exist, when, on examining the limb *adroitly,* no grating, or rubbing of the ends of the bones, are perceived.

The application of *vesicatories,* after the manner abovementioned, has been evidently serviceable since in instances, a few of which have occurred where there was a sluggish disposition in the part, or a tardiness, to unite, particularly in a case of a fracture of the fore-arm, (both bones broken,) which remained as loose or as disunited as at first, after several weeks, which was consolidated by means of repeated vesicatories. It will be understood, of course, that, in all the instances I have alluded to, the persons were free from disease of any kind. The vesicatories were repeated to five or six times, and more in some instances.

In a fracture of the thigh or leg, it is essential to the well-doing of the limb that the body of the patient be kept in the same position exactly, whether on the side or the back, as that on which the limb is laid, any occasional twisting in the body, during the process of the consolidation of the bone, tending to produce a distortion in the limb. This is a circumstance requiring, on the part of the surgeon, constant attention, in consequence of its irksomeness to the patient; and nothing but a strong impression of the necessity of it, in order to obtain a perfect cure, without distortion, can make the the patient comply with it. When it becomes necessary for the patient to lie on the side, he should be propped up by pillows, or otherwise, and there should be no inducement, by attendants or visitors, to incline him to the contrary side. Should any temporary motion or alteration in the body be required, the limb itself should be kept steady in its place by a firm pressure of an attendant's hand.

It will be understood, that what I have inculcated respecting the occasional adjustment of fractured limbs, arises from necessity. Unquestionably, the limb after being once *set*, as it is called, should be kept as quiet, or as free from motion, as possible.

In cases, however, where the ends of the bone do not readily coalesce, a gentle motion, or friction of the part, has been found to promote union.

It is essential in fractures of the thigh or leg, especially in the former, that the bed, or rather the mattress, be as near to a plain surface, and as little *yielding*, or free from hollowness, as possible.

In a fractured thigh, *two splints only* should not be trusted to, as is customary with many practitioners; *four* being required to ensure a good limb.

It will, of course, be necessary, that the leg, when placed in the fracture-box, as above mentioned, should be somewhat elevated by pillows, so as to allow of the *bent* or *relaxed position* of the knee, and the ham duly supported, or filled up underneath, provided the surgeon has not the kind of fracture-box, which admits itself of the necessary elevation.

These observations may appear superfluous, or too minute to some persons; I well know, however, that they are *all* essential to the formation or restoration of a good limb; and that a thigh or leg, more or less distorted, after a simple fracture, is no very extraordinary occurrence.

In a fracture of the fore-arm, particular attention is required in the adjustment of the splints, from time to time, to preserve its due form, that is, free from the least twist.

Dislocations.—These cases were very frequent occurrences, especially of the *Humerus*, and, unless neglected for a length of time before application, were easily enough reduced.

The *Dislocation of the Femur*, I consider to be a very rare occurrence, *one only* presenting itself during the whole of my residence in the Infirmary. This case had been, previously to its arrival, represented to me, by a gentleman of the faculty, as a fracture near the hip-joint; I examined it, found it to be a dislocation, and immediately reduced it. I suspect that the dislocation at the hip-joint has, in some instances, by inexperienced practitioners, been mistaken for a fracture, and the patient, in consequence, remained lame his whole life after.

This is the only instance of dislocation at the hip-joint which I have ever seen; and I apprehend that many a practitioner in extensive practice may have passed his life without meeting with one.

I know of another, indeed, in an elderly person, in whom the dislocation was mistaken for a fracture by an experienced surgeon, long since dead; and another, from credible report, under similar circumstances, both of whom were compelled to use crutches the remainder of their lives.

There are two or more varieties of this accident, viz. one in which the head of the thigh-bone is dislocated forwards and downwards, in which the thigh is *elongated;* and another in which it is dislocated upwards and outward, in which the thigh is *shortened:* hence it *naturally* follows, that, in conjunction with other circumstances, this latter variety is more liable to be mistaken for a *fracture* of the thigh-bone, in which the thigh is *shortened* likewise.

The case which presented itself to me was of the former kind,* and probably the others were of the latter kind.

With respect to very bad cases of *compound fractures,* and *compound dislocations,* no part of surgery, perhaps, requires more judgment as to the possibility of preserving a limb without sacrificing the life of the patient. In a bad *compound fracture of the joint,* it will be generally prudent to decide in the negative. Very much, indeed, depends upon the after-management of the patient, in which it unfortunately happens too frequently that the patient is lost by too great officiousness in the surgeon. Mild treatment of the limb, with quietness, a little appropriate medicine, of which *opium* should be the chief,—and appropriate regimen, judiciously administered, would effect a cure in cases which, for want of such treatment, terminate fatally.

Tonsils enlarged.—The method commonly used for removing these is by ligature; for effecting which various modes and various kinds of instruments have been contrived.

* The person I allude to in this instance is William Bradbury, of the parish of Horsepath, near Oxford; and the person with the *disunited fracture,* who was cured by repeated blisters, is Thomas Izzard, in St. Clement's, viz. the suburbs of Oxford.

Having

Having experienced myself, and noticed in others, considerable difficulty in conveying a ligature round the tonsil, and afterwards making a knot upon it by the most usual method, viz. by means of the instrument well known to every surgeon, and represented in Bell's Surgery, vol. iv. plate 38, fig. 2. The following improvement on that method suggested itself to me. I procured two instruments, each consisting of a stem with a small ring at the end of it of polished iron fixed in a handle of wood; these were threaded with a waxed ligature of convenient size. Holding one of these instruments in each hand, I applied the ring of the instrument in my *left* hand to the base of the tonsil, confining the ligature from slipping, by pressing it against the handle; then, with the instrument in my *right* hand, I conveyed the ligature, by means of the ring, round the tonsil, so as to meet that which I held fixed in my left hand. I next withdrew both the instruments from the two ends of the ligature, and, threading one of the instruments, with both ends of the ligature, I conveyed the ring of the instrument close to the base of the tonsil, an assistant confining it there, whilst I made a single noose of the ligature, hanging without side the mouth, and drew it so as to make the noose pass through the ring of the instrument to the tonsil; then, withdrawing the instrument, I drew the knot sufficiently tight, and afterwards a second knot upon the former. By this method, I succeeded in the first attempt of making the ligature. It is necessary that the ring in each instrument be *sufficiently large, only*, to admit of the first-made noose passing readily through it.

It is sometimes necessary, in order to remove the tonsil, provided the first ligature be not sufficient completely to effect it, as may be sometimes the case when its base is very large or rigid, to renew the ligature after the same manner.

I am induced to consider the above improved method of removing *enlarged tonsils*, which was introduced by me* twelve

FROM THE EDITOR.

* We suggest to our ingenious correspondent, that a pair of curved scissars will effect the removal of a tonsil without the *pain* and delay consequent to the use of the ligature. The Editor has performed the operation of excision in many *varieties* of diseased and enlarged tonsils, and has found it infinitely preferable to the ligature, which in many cases *cannot* be applied; and in some has been attended with alarming erysipelatous inflammation, besides the pain it commonly produces. It is proper to add, that the

 hæmorrhagy twelve or thirteen years ago, as meriting the attention of others, for the following reasons: first, because I had observed a surgeon here, remarkable for his adroitness in this as well as every other operation he took in hand, in one instance, so completely baffled in repeated attempts to make the ligature in the ordinary way, as to be compelled to *give it in;* who, at my suggestion, succeeded instantly by my improved method, and which he ever afterwards pursued, with immediate success; and, secondly, because this method has been adopted generally, and constantly practised here, in preference to any other. The necessity of this operation was a familiar occurrence at the Radcliffe Infirmary.

CUPPING.—To those who do not succeed satisfactorily in this operation by the ordinary method, *with the lamp,* I would recommend the following method, which, after a great many experiments, I found to be the most perfect and easy method of exhausting the glasses of air. Having previously formed a convenient number of bell-shaped or conical pieces of bibulous paper, I dipped these, each immediately previous to using, the twisted handle excepted, into spirit of wine; then lighting it by a candle, I instantly placed it in the glass; then, giving the glass a rotatory motion for a few seconds, applied it over the scarified part, contrived that the lighted paper, the flame of which is extinguished at the instant the external air is excluded, shall not come in contact with, or in any manner incommode, the patient. I do not believe it possible, by any other method, to exhaust the glasses so completely of air, as by the one I have described.

The edge or lip of the cupping-glass should be slightly

---

hæmorrhagy which follows the operation is *always* very trifling. In some caustic has been employed with success; but the process is so tedious, that it can only be recommended where the use of the knife or scissars is not permitted.

One of the cases where excision was performed, was that of a young woman dismissed *as an incurable patient from the Infirmary,* and might possibly have been the patient of the surgeon above referred to; the ligature had been applied by passing a needle through the base of the gland, for the purpose of tying it in separate portions; instead of effecting this, it split the tonsil in two parts, and the patient was sent to London for the advice of the Editor. If Mr. Walker will take the pains to consult the records of the Hospital for the year ——, he will probably see the name of Eliz. Acres, the patient in question, who is now known by *one* of the surgeons of that institution to be perfectly recovered, contrary to his expectation.

wetted wetted with water before using, by means of a wetted cloth or sponge; and care should be taken that the paper be not so loaded with the spirit as to endanger its dropping.

The glass, when circumstances will admit of it, should be applied not vertically but horizontally.

It will be apparent, that the method I have recommended here is merely an improvement, if I may be allowed to call it so, of the method described in the System of Surgery, by Mr. Benjamin Bell, vol. iii. p. 160.

It will be observed, that the few professional improvements, as I consider them, which I now make public, and likewise some others which may follow, were first introduced into use in the Radcliffe Infirmary, and afterwards adopted in private practice many years ago, as will appear by referring to the dates. I lay a stress upon this circumstance, because my peculiar situation in the Infirmary, at that time, rendered such public communication, in my opinion, improper; and, moreover, as I understand, these improvements, originating in myself, have been long since adopted and pursued, but *imperfectly*, in various distant parts.

The improvements I principally allude to, at present, are, "the extraordinary efficacy of a new modification of *carrot poultice*, in sores and ulcers, especially such as are of a highly malignant nature; first introduced into practice here in the year 1795, but not published till the year 1806.* Likewise, on the efficacy of repeated *vesicatories*, in cases of fractures which have remained loose, or have not coalesced;" and "the certain and ready method of removing *enlarged tonsils;*" both forming a portion of this paper.

RICHARD WALKER.

*Oxford, Oct.* 8, 1814.

P. S.—I beg leave to correct an *erratum* which has occurred in my last paper. For the *note* at page 291, read—"The matter of cow-pox being apparently of a milder or less virulent nature than that of small-pox, the circumstance of a local pustule is more likely to happen in the former than in the latter disease."

The latter portion of the above *note* is, by mistake, placed at the latter part of the first paragraph in page 292, to which it bears no relation.

---

* A copy of this paper is given at page 140 of the Medical and Physical Journal, for February 1810. The Appendix respecting the supposed efficacy of *turnip poultice, in similar cases,* was introduced without my knowledge.

